# *Monarda didyma* Hydrolate Affects the Survival and the Behaviour of *Drosophila suzukii*

**DOI:** 10.3390/insects13030280

**Published:** 2022-03-11

**Authors:** Luca Finetti, Stefano Civolani, Daniele Mirandola, Lorenzo Benetti, Santolo Francati, Federica Albanese, Felicia Menicucci, Marco Michelozzi, Maria Grazia Bellardi, Maria Luisa Dindo, Giovanni Bernacchia

**Affiliations:** 1 Department of Life Science and Biotechnologies, University of Ferrara, Via Borsari 46, 44121 Ferrara, Italy; fntlcu1@unife.it (L.F.); mrndnl1@unife.it (D.M.); lorenzo.benetti@edu.unife.it (L.B.); 2 Department of Environmental Sciences and Prevention, University of Ferrara, Via Borsari 46, 44121 Ferrara, Italy; cvlsfn@unife.it; 3 Department of Agricultural and Food Sciences (DISTAL), University of Bologna, Viale Giuseppe Fanin, 40-50, 40127 Bologna, Italy; santolo.francati2@unibo.it (S.F.); mariagrazia.bellardi@unibo.it (M.G.B.); marialuisa.dindo@unibo.it (M.L.D.); 4 Department of Biomedical and Specialty Surgical Sciences, Section of Pharmacology, University of Ferrara, Via Borsari 46, 44121 Ferrara, Italy; lbnfrc@unife.it; 5 Institute of Chemistry of Organometallic Compounds, CNR, Via Madonna del Piano 10, 50019 Sesto Fiorentino, Italy; felicia.menicucci@iccom.cnr.it; 6 Institute of Biosciences and Bioresource, CNR, Via Madonna del Piano 10, 50019 Sesto Fiorentino, Italy; marco.michelozzi@cnr.it

**Keywords:** hydrolate, essential oils, monoterpenes, Lamiaceae, *Monarda didyma*, *Drosophila suzukii*, egg-laying assay, pest control, biopesticide

## Abstract

**Simple Summary:**

During the steam distillation of aromatic plants, two main fractions are usually obtained: the hydrophobic essential oils and the hydrophilic fraction commonly known as hydrolate (HY). The essential oils are largely used in several industry fields, including the agricultural industry as biopesticides. Residual HYs, instead, are often discarded as by-products of little or no value. Our research pointed out that also HYs have biological activity, suggesting their potential use in plant-based strategy for the pest control. In more detail, we investigated the insecticidal properties of the hydrolate from *Monarda didyma*, scarlet beebalm, towards *Drosophila suzukii*. Using specific molecular and behavioural assays, we showed that *M. didyma* hydrolate affected the fitness and behaviour of *D. suzukii*, providing new insights in the *D. suzukii* control strategies through *M. didyma* hydrolate.

**Abstract:**

*Drosophila suzukii* (Matsumara) is an herbivorous pest whose control in the field with conventional chemical is particularly difficult and has important drawbacks. Here, we investigated the insecticidal properties of hydrolate from *Monarda didyma*, scarlet beebalm, an aromatic herb in the Lamiaceae family. The identification of volatile organic compounds (VOCs) by CG–MS systems revealed that thymol (38%) and carvacrol (59%) were the most abundant VOCs in the hydrolate. *M. didyma* hydrolate did not show fumigant toxicity. Conversely, in contact assays, *M. didyma* hydrolate showed a LC_50_ of 5.03 µL mL^−1^, 48 h after the application on *D. suzukii* adults. Expression of detoxification genes increased in flies that survived the LC_50_ application. Furthermore, toxicity persisted for 7 days after the treatment in the survival evaluation. Artificial diet assays with 100 and 1000 µL mL^−1^ of *M. didyma* hydrolate resulted in a significant decrease in total food intake in both male and female *D. suzukii* adults. In addition, electropenetrography (EPG) showed that the *D. suzukii* females’ feeding behaviour was altered in hydrolate-treated diets. The hydrolate also caused a significant reduction in the number of eggs laid in two different oviposition assays. Overall, our findings provide a new perspective for the improvement of *D. suzukii* control strategies through *M. didyma* hydrolate.

## 1. Introduction

New and sustainable pest management strategies are needed to control the spread and damage of insect pests. In this scenario, pest control requires more and more the identification of natural and environmentally friendly compounds with insecticidal activity. Plant-derived essential oils (EOs) are known to be important sources of natural insecticides to use in pest control [1,2,3]. EOs have traditionally been used to repel flying insects and protect agriculture products [4,5,6], although with limited success [7,8]. However, several studies have explored the possibility to adopt EOs as insecticides, showing fumigant or direct contact toxicity [9,10,11,12].

*Drosophila suzukii* (Matsumura) (Diptera: Drosophilidae), or spotted wing *Drosophila* (SWD), has successfully spread to Europe and the USA, but also South America, Canada, and Africa, from its areas of origin (China, Taiwan, and Korea) [13,14,15,16]. Small red fruits (such as blackberries, raspberries, and cherries) are the most affected, but *D. suzukii* can attack other species, such as olives, spicebush, and mistletoe [17,18,19].

The *D. suzukii* control based on conventional insecticides has limitations because fruits near harvest cannot be treated [20] and it is not always effective [21,22]. For this reason, *D. suzukii* is an ideal candidate for testing natural compounds effective against this pest that can be used up to fruit harvest. Both Lamiaceae and Myrtaceae plant EOs have been shown to possess strong insecticidal action against *D. suzukii* [23,24], appearing effective in different ways [25]. For example, in a detailed study, Kim et al. [26] successfully tested 22 EOs as fumigants and contact insecticides against *D. suzukii*. Furthermore, Piperaceae plant EOs also showed toxicity towards *D. suzukii* [27]. Besides the direct insecticide activity, EOs have shown promising repellent effects as well as phagodeterrent properties [28,29,30]. Recently, monoterpenes such as thymol, carvacrol, and α-terpineol, which are major components of many EOs, were shown to affect *D. suzukii* behaviour and have insecticidal properties [31,32].

During the steam distillation of essential oils, two main fractions are usually obtained: the hydrophobic EOs and the hydrophilic fraction commonly known as hydrolate (HY) or hydrosol [33]. In the HY, the relative ratio of each terpenic molecule depends on its hydrophilic characteristics [34]. For this reason, the major components of an EO may not be the same present in the corresponding HY. However, several HYs possess antimicrobial and antifungal activity [33,35,36,37,38,39], but only a few studies have investigated their insecticidal action. Petrakis and colleagues [40] examined the possible use of HYs from *Origanum majorana* L., *Mentha pulegium* L., and *Melissa officinalis* L. against *Myzus persicae* Sulzer, showing that they altered the insect locomotion and fitness. Moreover, *M. pulegium* and *Mentha suaveolens* Ehrh. HYs induced mortality in the aphid *Toxoptera aurantia* (Fonscolombe) [41]. Only the HYs from *Solanum granuloso-leprosum* Dun. and *Ricinus communis* L. were tested as insecticides on Diptera species, but they appeared to have no effect against *Anastrepha fraterculus* (Wiedemann) and *Ceratitis capitata* (Wiedemann) pupae and adults [42].

In the last decade, scarlet beebalm, *Monarda didyma* L. (Lamiales: Lamiaceae), EO and HY have been tested for specific application in both humans and plants in order to study their main properties, such as psychopharmacological, anti-cancer, larvicidal, or nematocidal activities [43,44]. In addition, studies were carried out in 2019 and 2020 to verify for the first time the insecticide activity of *M. didyma* HY towards the whitefly *Trialuerodes vaporariorum* (Westwood), one of the most harmful and widespread pests of greenhouse crops. The results obtained showed that HY is more effective than two commercial insecticidal products used as controls [45,46].

In this study, the effect of *M. didyma* HY on *D. suzukii* physiology and fitness was assessed through molecular and behavioural assays.

## 2. Materials and Methods

### 2.1. Insect Rearing

*D. suzukii* was kindly provided by the Edmund Mach Foundation, Trento (Italy). Larvae and adults were reared on an artificial diet [47], at 22 ± 1 °C, 70% relative humidity, and 16 h light/8 h dark photoperiod.

### 2.2. Monarda didyma: Plant Material and HY Extraction

In 2017, *M. didyma* (voucher no. FPMO01) seedlings obtained from seeds in greenhouse were transplanted to field plots in April 2017 at the “Scarabelli-Ghini Agricultural Institute” of Imola (Bologna, Emilia-Romagna, Italy) (44°21′ N, 11°422′ E, 47 masl) and cultivated for the growing seasons with appropriate husbandry. Fresh aerial parts (around 70% leaves and 30% stems) were collected at the end of the growth season and immediately used for hydro-distillation at the Herb Garden of Casola-Valsenio (Ravenna) to obtain the HY. All plants were regularly monitored to control growth, pests, and diseases until blooming.

### 2.3. M. didyma HY Composition

Hydrosol extract was obtained by liquid–liquid extraction by mixing 0.5 mL of *M. didyma* HY with 0.5 mL of heptane using a mixer mill (Retsch-MM300, Haan, Germany) for 1 h at 4 cycles per second. Next, 300 μL of supernatant was centrifuged at 4000 rpm for 10 min at 10 °C in an Eppendorf centrifuge mod. 5810R (Westbury, NY, USA). The heptane extracts were then filtered with 0.45 μm polytetrafluoroethylene (PTFE) syringe filters and analysed using an Agilent 7820A gas chromatograph (GC) and a 5975C mass spectrometer (MS), all from Agilent Tech. (Palo Alto, CA, USA), by injecting 1 µL of hydrosol extract with a split/splitless injector operating in splitless. A Gerstel MPS2 XL autosampler equipped with liquid option was used. The chromatographic settings were as follows: injector in splitless mode set at 260 °C, J&W Innovax column (50 m, 0.25 mm i.d., 0.5 µm df); oven temperature program: initial temperature 40 °C for 1 min, then 5 °C min^−1^ until 200 °C, then 10 °C min^−1^ until 220 °C, then 30 °C min^−1^ until 260 °C, hold time 3 min. The mass spectrometer was operating with an electron ionisation of 70 eV, in scan mode in the *m*/*z* range 29–330, at three scans s^−1^. The identification of volatile organic compounds (VOC) was based on both peak matching with library spectral database (NIST 11), and kovats retention indices (KRI) were calculated from the retention data of the n-alkane (C6–C24) mixture standard and compared with those retrieved in the literature for the identified compounds. The amount of each monoterpene was expressed as percentage of total monoterpenes.

### 2.4. M. didyma HY Fumigant Toxicity Assay

The fumigant toxicity assay was performed as previously described [32] in a polyacrylic cylinder (16 cm in height, 3.5 cm inner diameter; 0.15 L^−1^ total volume). The *M. didyma* HY was diluted in water and then applied to a filter paper (2 cm × 2 cm) placed on the bottom lid of the cylinder, inside a small cage to prevent the direct contact with the flies. A total of 20 µL of five different *M. didyma* HY concentrations, ranging from 0.001 to 1000 µL mL^−1^, were tested along with negative control (water). *D. suzukii* adults (15 males and 15 females, 3 to 5 days after emergence) were placed inside the cylinder with cotton pads soaked in water and honey to prevent flies’ dehydration. The cylinder top and the bottom were then sealed with Parafilm. During the assay, the following conditions were maintained constant: temperature at 22 ± 1 °C, 16 h light/8 h dark, 70% relative humidity. The mortality was observed after 24 and 48 h and used to perform the LC_50_ and LC_90_ analyses. Treatments were replicated three times, with 30 flies per replication.

### 2.5. M. didyma HY Contact Toxicity Assay

A Potter spray tower (Burkard, Uxbridge, UK) was used to examine the *M. didyma* HY toxicity towards *D. suzukii* adults. *D. suzukii* adults (20 males and 20 females), 3 to 5 days after emergence, were anesthetised in ice and placed in a 9 cm diameter open Petri dish. A 2 mL solution containing six different concentrations of *M. didyma* HY, ranging between 0.001 and 1000 µL mL^−1^, was then sprayed (7 × 10^5^ Pascal) onto the Petri dish, which was then immediately closed with a coverslip presenting a mesh-covered opening to allow drying. A cotton pad soaked in water and honey was then inserted into the dish for flies’ survival. Water alone was sprayed as negative control. The assay was replicated three times with 40 flies per replicate, and the following conditions were maintained constant during the assay: temperature at 22 ± 1 °C, 16 h light/8 h dark, 70% relative humidity. The mortality was observed after 24 and 48 h and used to perform the LC_50_ and LC_90_ analyses.

### 2.6. Survival Assay

Adult flies (three replicates with 20 insects each) that survived after 48 h of contact exposure to the *M. didyma* HY LC_50_ were reared into new artificial diet vials and kept under controlled conditions (22 ± 1 °C, 16 h light/8 h dark, 70% relative humidity), changing the diet every day. The number of dead flies was recorded for 14 days to assess the survival and, therefore, the sub-lethal toxicity of *M. didyma* HY.

### 2.7. Gene Expression Analysis after M. didyma Treatment

Total RNA was extracted from *D. suzukii* adults (three replicates with 3 males and 3 females each) that survived the contact with the *M. didyma* HY LC_50_ concentration, or water alone as controls, using an EZ-10 spin column animal total RNA mini-prep kit (Bio Basic, Markham, ON, Canada). A total of 1 μg of RNA was treated with DNase I (New England Biolabs, Ipswich, MA, USA) and used for cDNA synthesis, carried out with a RevertAid First Strand cDNA Synthesis Kit (Thermo Fisher Scientific, Waltham, MA, USA) according to the manufacturer’s instructions. Real-time PCR was performed using a CFX Connect Real-Time PCR Detection System (Bio-Rad, Hercules, CA, USA) in a 12 μL reaction mixture containing 0.8 μL of total cDNA, 6 μL SsoAdvanced Universal SYBR Green Supermix (Bio-Rad, Hercules, CA, USA), 0.4 μL forward primer (10 μM), 0.4 μL reverse primer (10 μM), and 4.4 μL of nuclease-free water. Thermal cycling conditions were: 95 °C for 3 min, 40 cycles at 95 °C for 15 s, and 60 °C for 30 s. A melting-curve analysis from 55 °C to 95 °C was applied after the amplification protocol. The gene expression levels were quantified using the Livak method [48], and *AK* and *TBP* were used as reference housekeeping genes [49]. Gene-specific primers were designed for seven genes coding for different detoxification enzymes (*Cyp4e3*, *Cyp4g15*, *Cyp6a17*, *Cyp6d5-2*, *Gstd10*, *Gstz2*, *Est1*) (Table S1).

### 2.8. Dye-Labelling Food Intake Assay

Dye-labelling food intake quantification was performed as previously described [32]. A pool of 6 flies (females or males) were placed into a vial with 1 mL of dyed medium (2.5% (*w*/*v*) yeast, 2.5% (*w*/*v*) sucrose, 1% (*w*/*v*) agar, and 1% (*w*/*v*) Brilliant Blue FCF; Sigma Aldrich, St. Louis, MO, USA), and 100 μL of a solution containing 1000, 100, or 10 µL mL^−1^ of *M. didyma* HY was distributed on the surface. Water was tested as negative control. After 2 h of feeding, the flies were collected and frozen at −80 °C. The samples were then homogenised with a micropestle in 50 μL of 1% PBST (PBS with 1% of Triton-X) and centrifuged for 1 min at 12,000× *g* to clear the debris. The supernatant absorbance was measured at 630 nm on a label-free EnSight Multimode Plate Reader (Perkin Elmer, Waltham, MA, USA). The values obtained from flies fed with non-labelled food were used as a control and subtracted from the experimental readings. To determine the dye concentration for each fly homogenate, a standard curve was generated with serial dilutions of a 10 μL aliquot of the non-solid dye-labelled food added to 990 μL of 1% PBST. Three replicates (6 flies each) were performed for each sex group.

### 2.9. Feeding Behaviour by Electropenetrography (EPG)

**Figure 1:**
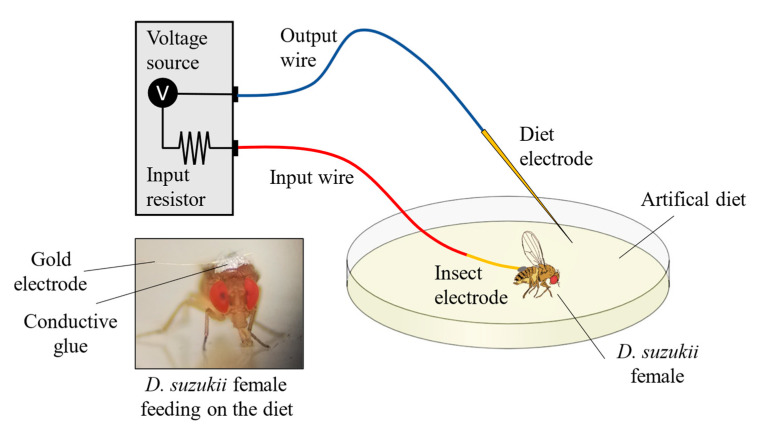
Electropenetrography (EPG) setup scheme used to analyse the *D. suzukii* feeding behaviour on artificial diets.

EPGs of female adult *D. suzukii* were performed as previously described [50]. Two *D. suzukii* females (20–23 flies in total, 3 to 5 days after eclosion) were placed in a 9 cm Petri dish covered with artificial diet (1% agar, 2.5% sucrose) surface-treated with either 1000 µL mL^−1^ *M. didyma* HY (140 µL per spray) or water (negative control) in the laboratory at 22 ± 1 °C, under artificial fluorescent light (4000 Lux) and a 16:8 light/dark photoperiod. EPGs were recorded for 2 h for both diets. Before each experiment, a fly was collected from the rearing colony and then immobilised in a chilled Petri dish. A thin gold electrode wire (18 μm diameter and 2 cm long) was glued to the dorsum of the insect by a water-based silver glue (Wageningen Agricultural University, Wageningen, The Netherlands). The gold electrode was connected to the amplifier by a long and thick copper wire. A second copper electrode was inserted in the artificial diet (Figure 1).

An EPG DC-type, Giga-4 model device was used (Wageningen Agricultural University, Wageningen, the Netherlands) with an input resistance of 1G Ω. After A/D conversion at 100 Hz (Di710 USB, Dataq, Akron, OH, USA), the *D. suzukii* EPG waveforms [50] were acquired and studied by Stylet+ software (for Windows; Wageningen Agricultural University, Wageningen, The Netherlands). A supplementary EPG experiment was performed using *D. suzukii* females previously exposed for 24 h to the LC_50_ *M. didyma* HY or water (control) in order to investigate if the exposure might affect the feeding behaviour. In both the EPG studies, the feeding phases (probing, non-probing, and dabbing) were individually analysed.

### 2.10. Laboratory Egg-Laying Assay

A laboratory egg-laying assay was performed in a 12-well plate containing 500 µL of artificial diet, used as substrate for oviposition (1% (*w*/*v*) agar, 2.5% (*w*/*v*) sucrose) and 100 µL of *M. didyma* HY solution containing 1000, 100, or 10 µL mL^−1^; water was used as a negative control. One anesthetised *D. suzukii* female was placed in each well for a total of 12 flies tested for each replicate, with four replicates in total (16 insects in total for each concentration tested). Each *D. suzukii* female, before performing the assay, was prevented from laying eggs for three days by using rear vials presenting a larvae-full surface that physiologically did not allow them to lay eggs [51]. The plate was then kept under controlled conditions (22 ± 1 °C, 16 h light/ 8 h dark, 70% of relative humidity), and the number of eggs laid was counted using a stereomicroscope after 24 h.

### 2.11. Cherry Oviposition Assay

The effects of *M. didyma* HY on *D. suzukii* females’ oviposition response were investigated in a dual choice assay. The experiment was performed in a net cage (70 × 30 × 30 cm) placed in the lab at 22 ± 1 °C, 70% relative humidity, and a 16 h light/8 h dark photoperiod. For each replicate, 25 *D. suzukii* females were placed in the cage along with 30 cherries divided in two groups. One group was evenly sprayed with 1 mL of *M. didyma* HY solution (100 or 1000 µL mL^−1^), or water as negative control, by Potter spray tower, as described above. The second group of cherries was left untreated. During the experimental period, the average temperatures in the cages ranged from a minimum of 17 °C during the night to a maximum of 24 °C during the day. After 48 h, the total number of eggs on each group of cherries was evaluated using a stereomicroscope. Three replicates (75 flies in total) were performed for each concentration.

### 2.12. Statistical Analyses

Concentration-mortality data were subjected to Probit analysis using POLO-plus software (LeOra Software Company, Northampton, UK). All other analyses were performed using GraphPad Prism software (version 6, San Diego, CA, USA). In EPG analysis data, all non-sequential parameters (waveform duration and number of events per individual insect) and sequential parameters (first probing phase and second non-probing duration) were analysed using the non-parametric ANOVA Mann–Whitney *U* test (*p* < 0.05) (software STATISTICA 6, StatSoft, Tulsa, OK, USA).

## 3. Results

### 3.1. M. didyma HY Composition

**Figure 2:**
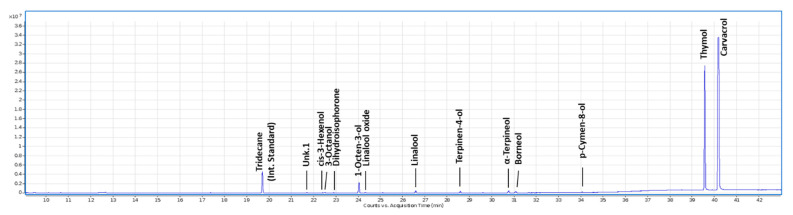
Total ion chromatogram (TIC) of monoterpenes and other volatile organic compounds present in *M. didyma* HY.

The GC–MS analysis revealed that the main component contained in the *M. didyma* HY was carvacrol (59.20% of total monoterpenes), followed by thymol (38.50% of total monoterpenes). The other minor oxygenated monoterpenes were linalool (0.97% of total monoterpenes), terpinen-4-ol (0.88% of total monoterpenes), and α-terpineol (0.44% of total monoterpenes) (Figure 2 and Figure S1).

### 3.2. M. didyma HY Fumigant and Contact Toxicity Assay

Fumigant assays showed that the *M. didyma* HY do not cause mortality on *D. suzukii,* regardless of the concentrations tested (from 0.001 to 1000 µL mL^−1^, data not shown). On the other hand, the Potter spray tower showed *M. didyma* HY toxicity by contact against *D. suzukii*. The LC_50_ values of the *M. didyma* HY were calculated 24 or 48 h upon treatment. At 24 h, the data were particularly dispersed and the LC_50_ and the LC_90_ could not be calculated (Table 1). At 48 h, *M. didyma* HY application showed a LC_50_ value of 5.03 µL mL^−1^.

### 3.3. Effect of M. didyma Contact Exposure on Survival and Detoxification Enzyme Expression

**Figure 3:**
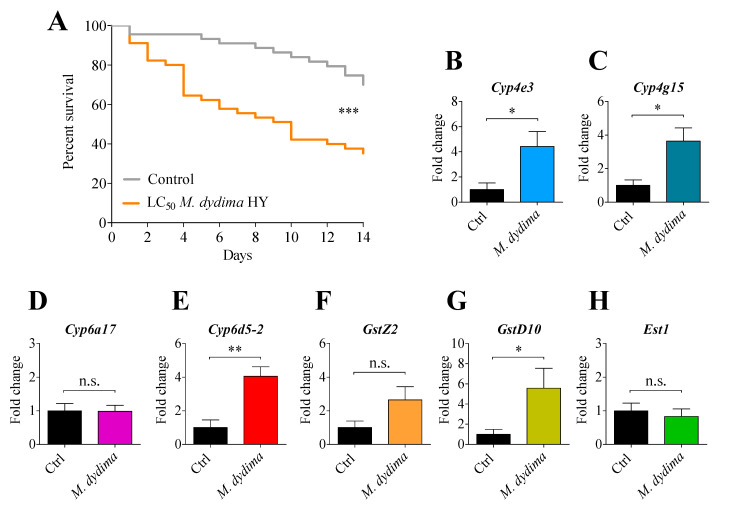
Effect of *M. didyma* contact exposure on survival and detoxification enzyme expression. Survival assay of *D. suzukii* adults after *M. didyma* HY LC_50_ exposure (**A**). Fifteen flies for each biological replicate were scored for survival, and three biological replicates were performed. For survival assay, statistical analyses were performed using the log-rank test. *** *p* < 0.001. *D. suzukii* metabolic gene expression levels after 48 h of exposure to the *M. didyma* HY LC_50_ (**B**–**H**). Data represent means ± SEM of three independent experiments performed in triplicate. n.s. not significant; * *p* < 0.05, ** *p* < 0.01 vs. control according to Student’s *t*-test.

The treatment with the *M. didyma* HY LC_50_ (5.03 µL mL^−1^) negatively affected the *D. suzukii* life duration (Figure 3, panel A). In particular, *M. didyma* HY caused a prolonged mortality on hydrolate-treated *D. suzukii* as compared to water treatment. Mortality was ≈40% higher in comparison to control flies within five days upon treatment.

Given the higher mortality in flies exposed to *M. didyma*, we investigated whether the treatment might also trigger biomolecular responses such as the detoxifying systems of *D. suzukii*. To test this hypothesis, we evaluated the mRNA abundance of a series of genes coding for detoxification enzymes belonging to the P450 monoxygenase (CYPs) [52], the glutathione-S-transferase (GSTs) [53], and the esterase superfamilies (EST) [54]. *D. suzukii* flies that survived the LC_50_ *M. didyma* HY treatment showed an upregulation of some P450 genes, such as *cyp4e3* (Figure 3, panel B), *cyp4g15* (Figure 3, panel C), and *cyp6d5-2* (Figure 3, panel E), as well as a GST-coding gene, *gstD10* (Figure 3, panel G). This evidence suggests that the *M. didyma* HY contact-treatment not only affects *D. suzukii* survival but it also upregulates detoxification genes, therefore suggesting an interference with the general metabolism and gene expression profiles of the insect.

### 3.4. Dye-Labelling Food Intake Assay

**Figure 4:**
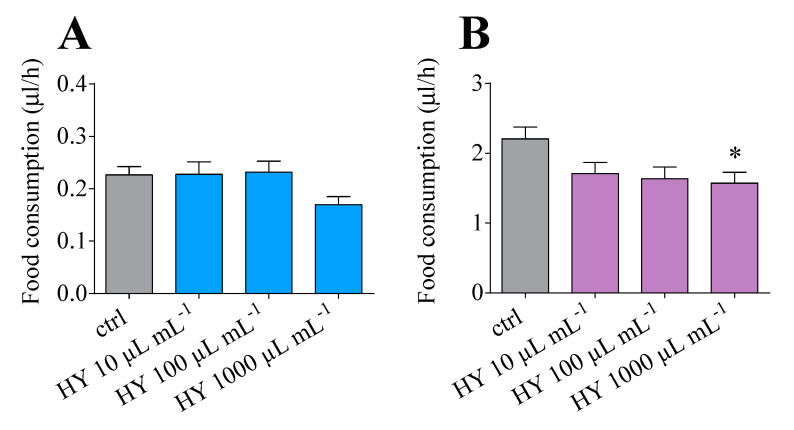
Food intake in *D. suzukii* adults after 48 h feeding on diet treated with *M. didyma* HY LC_50_. (**A**) Food consumption, measured as µL of diet per hour, of *D. suzukii* males. (**B**) *D. suzukii* females. Data shown are the means ± SEM of four independent biological replicates. * *p* < 0.05 vs. control according to one-way ANOVA followed by Dunnett’s test for multiple comparisons.

To further characterise the effect of *M. didyma* HY on *D. suzukii*, we tested the putative phagodeterrent activity with a food intake assay. Male and female flies were fed with a dye-labelled diet, containing different quantities of *M. didyma* HY, and the amount of food ingested per hour was measured. A significantly lower food intake was observed for the females only in the diet treated by 1000 µL mL^−1^ *M. didyma* HY (Figure 4, panel B), while male flies did not show any variation in their feeding behaviour (Figure 4, panel A). In this scenario, females appeared more susceptible than male flies to the *M. didyma* HY phagodeterrent activity.

### 3.5. Feeding Behaviour by Electrical Penetration Graph (EPG)

*D. suzukii* feeding behaviour was also studied by EPG. Table 2 shows a comparison of probing and non-probing phases of *D. suzukii* females recorded by EPG on both 1000 µL mL^−1^ *M. didyma* HY-treated and control artificial diets. No significant differences between the two diets were observed in terms of mean ingestion duration per insect and mean duration of each ingestion event. However, flies feeding on *M. didyma* HY-treated diets showed a significant lower number of ingestion events. In particular, the mean number of ingestion events in *M. didyma* HY was 94.46 ± 11.07, while the control reached a mean number of 157.91 ± 25.02 (*p* = 0.04). Moreover, the mean duration of each non-probing event was significantly higher in *M. didyma* HY-treated diet (1.26 ± 0.18 min vs. 0.90 ± 0.17 min, *p* = 0.02), while the dabbing, a subfamily of a probing behaviour, showed a statistically longer mean duration of each event in *D. suzukii* on the *M. didyma* HY-treated diet.

The EPG feeding measurements were also performed on females previously fed for 24 h on diets with or without *M. didyma* HY 1000 µL mL^−1^. No significant differences were observed between *D. suzukii* females exposed to *M. didyma* HY or to control in both probing and non-probing phases. However, flies showed a significant lower mean duration of the dabbing events when previously fed for 24 h on a diet containing *M. didyma* HY (Table S2).

### 3.6. Egg-Laying Assays

**Figure 5:**
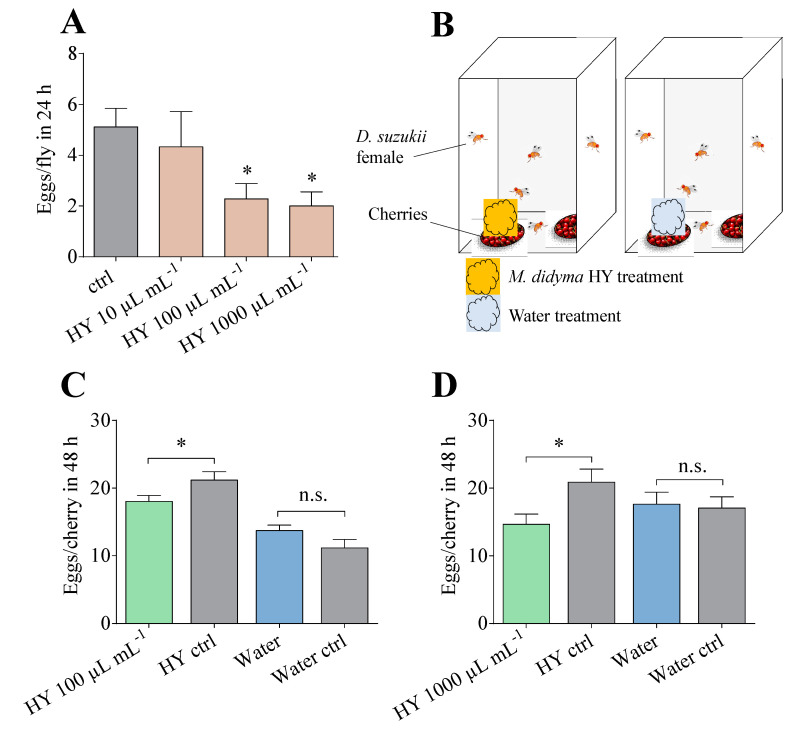
Effect of *M. didyma* HY on *D. suzukii* egg-laying. (**A**) The eggs (mean ± SEM) laid in 48 h by a single *D. suzukii* female with or without different amounts of *M. didyma* HY in the diet. * *p* < 0.05 according to one-way ANOVA followed by Dunnett’s test for multiple comparisons. (**B**) The schematic representation of the cherry oviposition assay performed with *D. suzukii* female adults. (**C**,**D**) The eggs (mean ± SEM) laid on one cherry with or without 100 µL mL^−1^ (**C**) and 1000 µL mL^−1^ (**D**) *M. didyma* HY. n.s. not significant; * *p* < 0.05 according to Student’s *t*-test.

The potential negative effect of *M. didyma* HY on *D. suzukii* oviposition was assessed in a laboratory assay. *D. suzukii* females laid significantly less eggs in 100 and 1000 µL mL^−1^ *M. didyma* HY-treated artificial diets (Figure 5, panel A). In particular, 1000 µL mL^−1^ of *M. didyma* HY reduced the eggs laid by 61% in comparison to the control, while 100 µL mL^−1^ of *M. didyma* HY was able to reduce the eggs laid by 56%.

Similar egg-laying experiments were also performed on cherries treated with both concentrations (100 µL mL^−1^ and 1000 µL mL^−1^) of *M. didyma* HY (Figure 5, panel B). During the 48 h period, a significant reduction (20% with 100 µL mL^−1^ and 32% with 1000 µL mL^−1^ *M. didyma* HY) in the number of eggs laid was measured on *M. didyma* HY-treated fruits as compared to the untreated cherries present in the same cage (Figure 5, panels C and D). The treatment with water did not alter the *D. suzukii* egg-laying behaviour, suggesting a direct effect of *M. didyma* HY on oviposition.

## 4. Discussion

The EOs obtained from various medicinal and aromatic plants have raised great attention in the scientific community and represent the “gold standard” natural products for several applications [9]. Residual hydrolates, instead, are often discarded as by-products of little or no value. In fact, they usually find application only in the cosmetic industry [55]. However, our research pointed out that also HYs have biological activity, suggesting their potential use in plant-based strategy for *D. suzukii* control.

Concentrations of thymol and carvacrol usually vary among different Lamiaceae plant organs [56]. In particular, thymol is more abundant in flowers than leaves in *Monarda* species (the stems are the poorest) and therefore the HY aromatic profile appears to be influenced by the relative proportion of leaves, flowers, and stems used for the extraction [57]. By GC–MS characterisation, we found that thymol and carvacrol were the most abundant monoterpenes composing *M. didyma* HY extracted from the flowering parts, covering together 97.6% of the total monoterpenes. Di Vito and colleagues [34] observed that the most representative monoterpenes in the *M. didyma* HY obtained from the whole plant were thymol and carvacrol, accounting for 83%. These two monoterpenes were also the most abundant in the EO, even for both concentrations. On the other hand, another study reported that the main monoterpenes in *M. didyma* flowers were thymol and linalool, with almost no carvacrol [58].

Although the absolute number of terpenes can be affected by abiotic factors, the relative contents of constitutive monoterpenes (monoterpene profile) in mature tissues have been shown to be under strong genetic control and very weakly affected by abiotic factors [59,60,61,62]. Terpene markers have been largely used in plant chemotaxonomy to characterise different species, population within the species, hybrids, families, and clones [63]. For this reason, it is important to consider plant chemotypes, characterised by a specific terpene profile, as source for biopesticide. Indeed, the variation in *M. didyma* HY volatile compound profile might affect the efficacy towards insect pests.

The possibility to use plant-based chemicals as integrated pest management (IPM) solutions towards *D. suzukii* is not a novelty [15,64]. In fact, it has been observed that *D. suzukii* is susceptible to the toxic action of EOs and their components, in particular to limonene, carvacrol, thymol, and eugenol [23,25,26,31,32,65]. On the other hand, although HYs are partly composed by these terpene compounds, they had never been tested as putative biopesticide against this pest.

When tested as a volatile in a fumigant assay, the *M. didyma* HY did not show any toxic effect toward adult *D. suzukii*. As a matter of fact, monoterpenes are present in the HY at a low dosage (no more than 1 g L^−1^) [34], and thus it might be that they are not sufficient to induce mortality of the target insect in this stage. On the other hand, when tested by contact, the *M. didyma* HY induced mortality both 24 and 48 h after the application with a quite low LC_50_. A different efficacy in inducing mortality between contact and fumigant exposures was also reported in *D. suzukii* for several essential oils [25,26].

The contact exposure of the LC_50_ *M. didyma* HY showed both rapid (observed in the first 48 h after treatment) and persisted toxicity (observed in the 14 days following treatment), with a mortality peak five days after the treatment. HYs are in fact composed by many molecules, each acting on different targets that are not yet well characterised. The monoterpenes, for instance, are thought to be able to interact with the GABA receptor [66], the acetylcholinesterase [67], and the tyramine/octopamine system [68]. Thus, we are led to assume that *M. didyma* HY-mediated mortality in *D. suzukii* might be caused by a synergy of multiple molecules and multiple targets recognised. The observed variations in the expression level of genes encoding P450 enzymes (*Cyp4e3*, *Cyp6d5-2*, and *Cyp4g15*) and GST (*GstD10*) would somehow strengthen this assumption so that different pathways and physiological aspects of the flies might be affected by the *M. didyma* HY. The Cyp4e3 enzyme is implicated in the *D. melanogaster* tolerance to permethrin toxicity feeding-mediated and the Cyp6d5 enzyme showed a higher expression in antennae of *D. melanogaster* exposed to geraniol acetate and in midgut after xenobiotic treatments [69,70,71]. The gene *Cyp4g15* codifying for the relative enzyme has been observed as being overexpressed in imidacloprid-resistant *Diaphorina citri* populations [72]. Furthermore, the GstD10 enzyme is implicated in the insecticide malathion detoxificarion in *Bactrocera dorsalis* [73]. Thus, the contact exposure of flies to *M. didyma* LC_50_ activated pathways known to be involved in the responses to toxic molecules, such as synthetic insectidices or monoterpenes.

In addition to their insecticidal properties, the monoterpenes seem to be able to modulate several aspects of *D. suzukii* behaviour and physiology [32], as well as act as repellents [29,74,75]. In our hands, *M. didyma* HY appeared able to alter important *D. suzukii* activities, such as feeding and oviposition. It was recently observed that *M. didyma* EO is able to decrease both the number of eggs laid and the number of eggs hatched in the nematode *Meloidogyne incognita* [76]. Furthermore, thymol and carvacrol, the main monoterpenes present in the *M. didyma* HY, exerted repellent and deterrent activity on *D. suzukii* [77] as well as on *Callosobruchus maculatus* F., *Anopheles arabiensis* Patton, and *Frankliniella occidentalis* Pergande [78,79,80]. However, it remains to be demonstrated as to whether the observed alterations in *D. suzukii* oviposition and feeding are caused by a repellent rather than a phagodeterrent effect. In the EPG analysis, we observed shorter probing events in the presence of *M. didyma* compared to the controls, possibly suggesting phagodeterrent properties that make the food sources and oviposition substrate less suitable to the insects.

Overall, the goal of this study was to investigate whether *M. didyma* HY has the potential to be successfully employed in *D. suzukii* control. The results, especially the oviposition experiments, suggest that this by-product of EO distillation (cheap and with low impact on the environment and human health) might be recycled in formulations for sustainable crop protection against *D. suzukii,* with the final aim of limiting the use of conventional pesticides. More experiments will allow for better defining the feasibility of this approach, by studying, for example, *M. didyma* HY efficiency on other fruits in the laboratory as well as in the field. Side effects on non-target species will be also the topic of future studies. This important aspect needs to be considered also in cases of non-conventional pesticides, including microbial insecticides [81], plant-derived EOs, HYs, and their components [82]. On the other hand, *M. didyma* HY might also be used to enhance the effect of other insecticides. In fact, it is reported that EOs can potentiate the toxicity of some insecticides [83,84]; therefore, *M. didyma* HY could also be used along with other insecticides to control damages caused to crops.

## Figures and Tables

**Table 1:** Contact LC_50_ and LC_90_ for *M. didyma* HY against *D. suzukii* adults. LC_50_ and LC_90_ values are means plus 95% confidence interval. Bold indicates the LC_50_ value used in physiological and behavioural experiments.

*M. didyma* HY	Slope ± s.d	LC_50_ (95% CI)	LC_90_ (95% CI)	Heterogeneity	χ^2^
24 h	0.974 ± 0.099	/	/	8.24	/
48 h	0.986 ± 0.097	5.03 µL mL^−1^ (2.08–10.11)	100.04 µL mL^−1^ (36.28–274.74)	2.83	11.514

**Table 2:** Comparison of probing, non-probing, and dabbing phases (mean ± SEM) measured by EPG on *D. suzukii* females feeding on untreated and *M. didyma* HY-treated diets. n.s. = not significant at *p* < 0.05. * *p* < 0.05 according to non-parametric ANOVA Mann–Whitney *U* test.

**Probing**	**Mean Probing Duration per Insect (min)**	**Mean Number of Probing Events**	**Mean Duration of Probing Event (min)**
1000 µL mL^−1^ HY	8.85 ± 1.75	94.46 ± 11.07	0.10 ± 0.02
Control	12.48 ± 2.22	157.91 ± 25.02	0.10 ± 0.02
Statistical analysis	n.s.	*	n.s.
**Non-Probing**	**Mean Non-Probing Duration per Insect (min)**	**Mean Number of Non-Probing Events**	**Mean Duration of Non-Probing Event (min)**
1000 µL mL^−1^ HY	111.09 ± 1.74	122.42 ± 14.09	1.26 ± 0.18
Control	107.29 ± 2.21	197.39 ± 30.37	0.90 ± 0.17
Statistical analysis	n.s.	*	*
**Dabbing**	**Mean Dabbing Duration per Insect (s)**	**Mean Number of Dabbing Events**	**Mean Duration of Dabbing Event (s)**
1000 µL mL^−1^ HY	9.31 ± 3.82	27.22 ± 4.68	0.50 ± 0.29
Control	10.17 ± 2.24	37.83 ± 8.42	0.27 ± 0.02
Statistical analysis	n.s.	n.s.	*

## Data Availability

The datasets generated and analysed during the current study are available within the article and its Supplementary Materials, as well as from the corresponding author on reasonable request.

## Supplementary Materials

The following supporting information can be downloaded at https://www.mdpi.com/article/10.3390/insects13030280/s1, Table S1: Primer used in this study; Table S2: Comparison of probing, non-probing and dabbing phases (mean ± SEM) measured by EPG on *D. suzukii* females left for 24 h on both untreated and HY treated diets. n.s. = not significant at *p* < 0.05. * *p* < 0.05 according to non-parametric ANOVA Mann–Whitney U test; Figure S1: Magnification of Figure 2 of the total ion chromatogram (TIC) of main monoterpenes and other volatile organic compounds present in *M. didyma* HY.

## References

[1] Robu V., Covaci G., Popescu I.M. (2015). The use of essential oils in organic farming. Res. J. Agric. Sci..

[2] Isman M.B. (2020). Botanical insecticides in the twenty-first century—Fulfilling their promise?. Annu. Rev. Entomol..

[3] Chang Y., Harmon P.F., Treadwell D.D., Carrillo D., Sarkhosh A., Brecht J.K. (2022). Biocontrol potential of essential oils in organic horticulture systems: From farm to fork. Front. Nutr..

[4] Sarac A., Tunc I. (1995). Toxicity of essential oil vapours to stored-product insects. Z. Pflanzenkrankh. Pflanzenschutz.

[5] Shaaya E., Kostjukovsky M., Ishaaya I., Degheele D. (1998). Efficacy of phyto-oils as contact insecticides and fumigants for the control of stored-product insects. Insecticides with Novel Modes of Action. Mechanism and Application.

[6] Isman M.B. (2006). Botanical insecticides, deterrents, and repellents in modern agriculture and an increasingly regulated world. Annu. Rev. Entomol..

[7] Haddi K., Turchen L.M., Viteri Jumbo L.O., Guedes R.N., Pereira E.J., Aguiar R.W., Oliveira E.E. (2020). Rethinking biorational insecticides for pest management: Unintended effects and consequences. Pest Manag. Sci..

[8] Turchen L.M., Cosme-Júnior L., Guedes R.N.C. (2020). Plant-derived insecticides under meta-analyses: Status, biases, and knowledge gaps. Insects.

[9] Bakkali F., Averbeck S., Averbeck D., Idaomar M. (2008). Biological effects of essential oils—A review. Food Chem. Toxicol..

[10] Werdin González J.O., Gutiérrez M.M., Murray A.P., Ferrero A.A. (2010). Biological activity of essential oils from *Aloysia polystachya* and *Aloysia citriodora* (Verbenaceae) against the soybean pest *Nezara viridula* (Hemiptera: Pentatomidae). Nat. Prod. Commun..

[11] Werdin González J.O., Gutiérrez M.M., Murray A.P., Ferrero A.A. (2011). Composition and biological activity of essential oils from Labiatae against *Nezara viridula* (Hemiptera: Pentatomidae) soybean pest. Pest Manag. Sci..

[12] Brügger B.P., Martínez L.C., Plata-Rueda A., Castro B., Soares M.A., Wilcken C.F., Carvalho A.G., Serrão J.E., Zanuncio J.C. (2019). Bioactivity of the *Cymbopogon citratus* (Poaceae) essential oil and its terpenoid constituents on the predatory bug, *Podisus nigrispinus* (Heteroptera: Pentatomidae). Sci. Rep..

[13] Walsh D.B., Bolda M.P., Goodhue R.E., Dreves A.J., Lee J.C., Bruck D.J., Walton V.M., O’neal S.D., Zalom F.G. (2011). *Drosophila suzukii* (Diptera: Drosophilidae): Invasive pest of ripening soft fruit expanding its geographic range and damage potential. J. Integr. Pest Manag..

[14] Asplen M.K., Anfora G., Biondi A., Choi D.S., Chu D., Daane K.M., Desneux N. (2015). Invasion biology of spotted wing Drosophila (*Drosophila suzukii*): A global perspective and future priorities. J. Pest Sci..

[15] Tait G., Mermer S., Stockton D., Lee J., Avosani S., Abrieux A., Anfora G., Beers E., Biondi A., Burrack H. (2021). *Drosophila suzukii* (Diptera: Drosophilidae): A decade of research towards a sustainable integrated pest management program. J. Econ. Entomol..

[16] Cini A., Ioriatti C., Anfora G. (2012). A review of the invasion of *Drosophila suzukii* in Europe and a draft research agenda for integrated pest management. Bull. Insectol..

[17] Lee J.C., Bruck D.J., Curry H., Edwards D., Haviland D.R., Van Steenwyk R.A., Yorgey B.M. (2011). The susceptibility of small fruits and cherries to the spotted-wing Drosophila, *Drosophila suzukii*. Pest Manag. Sci..

[18] Briem F., Eben A., Gross J., Vogt H. (2016). An invader supported by a parasite: Mistletoe berries as a host for food and reproduction of Spotted Wing Drosophila in early spring. J. Pest Sci..

[19] Kenis M., Tonina L., Eschen R., van der Sluis B., Sancassani M., Mori N., Haye T., Helsen H. (2016). Non-crop plants used as host by *Drosophila suzukii* in Europe. J. Pest Sci..

[20] Haye T., Girod P., Cuthbertson A.G.S., Wang X.G., Daane K.M., Hoelmer K.A., Baroffio C., Zhang J.P., Desneux N. (2016). Current SWD IPM tactics and their practical implementation in fruit crops across different regions around the world. J. Pest Sci..

[21] Civolani S., Vaccari G., Caruso S., Finetti L., Bernacchia G., Chicca M., Cassanelli S. (2021). Evaluation of insecticide adaptive response in Italian population of *Drosophila suzukii*. Bull. Insectol..

[22] Sial A.A., Roubos C.R., Gautam B.K., Fanning P.D., Van Timmeren S., Spies J., Petran A., Rogers M.A., Liburd Q.E., Little B.A. (2019). Evaluation of organic insecticides for management of spotted-wing drosophila (*Drosophila suzukii*) in berry crops. J. Appl. Entomol..

[23] Park C.G., Jang M., Yoon K.A., Kim J. (2016). Insecticidal and acetylcholinesterase inhibitory activities of Lamiaceae plant essential oils and their major components against *Drosophila suzukii* (Diptera: Drosophilidae). Ind. Crops Prod..

[24] Park C., Jang M., Shin E., Kim J. (2017). Myrtaceae plant essential oils and their β-triketone components as insecticides against *Drosophila suzukii*. Molecules.

[25] Dam D., Molitor D., Beyer M. (2019). Natural compounds for controlling *Drosophila suzukii*. A review. Agron. Sustain. Dev..

[26] Kim J., Jang M., Shin E., Kim J., Lee S.H., Park C.G. (2016). Fumigant and contact toxicity of 22 wooden essential oils and their major components against *Drosophila suzukii* (Diptera: Drosophilidae). Pestic. Biochem. Physiol..

[27] de Souza M.T., de Souza M.T., Bernardi D., Krinski D., de Melo D.J., da Costa Oliveira D., Rakes M., Zarbin P.H.G., de Noronha Sales Maia B.H.L., Zawadneak M.A.C. (2020). Chemical composition of essential oils of selected species of Piper and their insecticidal activity against *Drosophila suzukii* and *Trichopria anastrephae*. Environ. Sci. Pollut. Res..

[28] Erland L.A.E., Rheault M.R., Mahmoud S.S. (2015). Insecticidal and oviposition deterrent effects of essential oils and their constituents against the invasive pest *Drosophila suzukii* (Matsumara) (Diptera:Drosophilidae). Crop Prot..

[29] Renkema J.M., Buitenhuis R., Hallett R.H. (2017). Reduced *Drosophila suzukii* infestation in berries using deterrent compounds and laminate polymer flakes. Insects.

[30] Wallingford A., Helsen S.P., Cha D.H., Loeb G.M. (2016). Behavioral response of spotted-wing drosophila, *Drosophila suzukii* Matsumara, to aversive odors and a potential oviposition deterrent in the filed: *Drosophila suzukii* deterrents. Pest Manag. Sci..

[31] Finetti L., Ferrari F., Calò G., Cassanelli S., De Bastiani M., Civolani S., Bernacchia G. (2020). Modulation of *Drosophila suzukii* type 1 tyramine receptor (DsTAR1) by monoterpenes: A potential new target for next generation biopesticides. Pestic. Biochem. Physiol..

[32] Finetti L., Tiedeman L., Zhang X., Civolani S., Bernacchia G., Roeder T. (2021). Monoterpenes alter TAR1-driven physiology in *Drosophila* species. J. Exp. Biol..

[33] Acimovic M.G., Teševic V.V., Smiljanic K.T., Cvetkovic M.T., Stankovic J.M., Kiprovski B.M., Sikora V.S. (2020). Hydrolates—Byproducts of essential oil distillation: Chemical composition, biological activity and potential uses. Adv. Mater. Technol..

[34] Di Vito M., Smolka A., Proto M.R., Barbanti L., Gelmini F., Napoli E., Bellardi M.G., Mattarelli P., Beretta G., Sanguinetti M. (2021). Is the antimicrobial activity of hydrolates lower than that of essential oils?. Antibiotics.

[35] Sagdic O., Ozcan M. (2003). Antibacterial activity of Turkish spice hydrosols. Food Control.

[36] Rabha B., Gopalakrishnam R., Baruah I., Singh L. (2012). Larvicidal activity of some essential oil hydrolates against dengue and filariasis vectors. J. Med. Res..

[37] Wang T.H., Hsia S.M., Wu C.H., Ko S.Y., Chen M.Y., Shis Y.H., Shien T.H., Chuang L.C., Wu C.Y. (2016). Evaluation of the antibacterial potential of liquid and vapor phase phenolic essential oil compounds against oral microorganisms. PLoS ONE.

[38] Garzoli S., Petralito S., Ovidi E., Turchetti G., Laghezza Masci V., Tiezzi A., Trilli J., Cesa S., Antonietta Casadei M., Giacomello P. (2020). *Lavandula x intermedia* oil and hydrolate: Evaluation of chemical composition and antibacterial activity before and after formulation in nanoemulsion. Ind. Crops Prod..

[39] Dindo M.L., Modesto M., Rossi C., Di Vito M., Burgio G., Barbanti L., Mattarelli P. (2021). *Monarda fistulosa* hydrolate as antimicrobial agent in articial media for the in vitro rearing of the tachidin parasitoid *Exorist larvarum*. Entomol. Exp. Appl..

[40] Petrakis E.A., Kimbaris A.C., Lykouressis D.P., Polissiou M.G., Ch Perdikis D. (2015). Hydrosols evaluation in pest control: Insecticidal and settling inhibition potential against *Myzus persicae* (Sulzer). J. Appl. Entomol..

[41] Zekri N., Handaq N., El Caidi A., Zair T., El Belghiti M.A. (2016). Insecticidal effect of *Mentha pulegium L*. and *Mentha suaveolens* Ehrh. hydrosols against a pest of citrus, *Toxoptera aurantia* (Aphididae). Res. Chem. Intermed..

[42] Oviedo A., Van Nieuwenhove G., Van Nieuwenhove C., Rull J. (2017). Biopesticide effects on pupae and adult mortality of *Anastrepha fraterculus* and *Ceratitis capitata* (Diptera: Tephritidae). Austral Entomol..

[43] Mattarelli P., Epifano F., Minardi P., Di Vito M., Modesto M., Barbanti L., Bellardi M.G. (2017). Chemical composition and antimicrobial activity of essential oils from aerial parts of *Monarda didyma* and *Monarda fistulosa* cultivated in Italy. J. Essent. Oil-Bear. Plants.

[44] Laquale S., Avato P., Argentieri M.P., Bellardi M.G., D’Addabbo T. (2018). Nematotoxic activity of essential oils from *Monarda* species. J. Pest Sci..

[45] Mariani V., Turci S., Michelozzi M., Pollini A., Bellardi M.G. (2019). Idrolati di *Monarda didyma* per la difesa delle piante dalle infestazioni di *Trialeurodes vaporariorum*. Natural 1.

[46] Mariani V., Turci S., Pollini A., Biffi S., Cencetti G., Bellardi M.G. (2020). Prove preliminari di efficacia insetticida dell’idrolato di *Monarda didyma* contro *Trialeurodes vaporariorum*. Atti Giornate Fitopatologiche.

[47] Dalton D.T., Walton V.M., Shearer P.W., Walsh D.B., Caprile J., Isaacs R. (2011). Laboratory survival of *Drosophila suzukii* under simulated winter conditions of the Pacific Northwest and seasonal field trapping in five primary regions of small and stone fruit production in the United States. Pest Manag. Sci..

[48] Livak K.J., Schmittgen T.D. (2001). Analysis of relative gene expression data using real-time quantitative PCR and the 2(-Delta Delta C(T)) method. Methods.

[49] Zhai Y., Lin Q., Zhou X., Zhang X., Liu T., Yu Y. (2014). Identification and validation of reference genes for quantitative real-time PCR in *Drosophila suzukii* (Diptera: Drosophilidae). PLoS ONE.

[50] Guedes R.N.C., Cervantes F.A., Backus E.A., Walse S.S. (2019). Electropenetrography of spotted wing drosophila (*Drosophila suzukii*) on pesticide-treated strawberry. J. Pest Sci..

[51] Gou B., Zhu E., He R., Stern U., Yang C.-H. (2016). High throughput assay to examine egg-laying preferences of individual *Drosophila melanogaster*. J. Vis. Exp..

[52] Bergé J.B., Feyereisen R., Amichot M. (1998). Cytochrome P450 monooxygenases and insecticide resistance in insects. Philos. Trans. R. Soc. B.

[53] Tu C.P., Akgül B. (2005). *Drosophila* glutathione S-transferases. Methods Enzymol..

[54] Montella I.R., Schama R., Valle D. (2012). The classification of esterases: An important gene family involved in insecticide resistance-a review. Memórias Inst. Oswaldo Cruz.

[55] Sarkic A., Stappen I. (2018). Essential oils and their single compounds in cosmetics—A critical review. Molecules.

[56] Naghdi Badi H., Abdollahi M., Mehrafarin A., Ghorbanpour M., Tolyat M., Qaderi A., Ghiaci Yekta M. (2017). An overview on two valuable natural and bioactive compounds, thymol and carvacrol, in medicinal plants. J. Med. Plants.

[57] Kapelev K.G. (1976). Results of introducing Monarda as an essential oil plant. Byulleten’ Gos. Nikitsk. Bot. Sada.

[58] Marchioni I., Najar B., Ruffoni B., Copetta A., Pistelli L., Pistelli L. (2020). Bioactive compounds and aroma profile of some Lamiaceae edible flowers. Plants.

[59] Baradat P., Marpeau A., Walter J., Müller-Starck G., Ziehe M. (1991). Terpene markers. Genetic Variation in European Populations of Forest Trees.

[60] Hanover J.W. (1992). Applications of terpene analysis in forest genetics. New For..

[61] Langenheim J.H. (1994). Higher plant terpenoids: A phytocentric overview of their ecological roles. J. Chem. Ecol..

[62] Besser K., Harper A., Welsby N., Schauvinhold I., Slocombe S., Li Y., Dixon R.A., Broun P. (2009). Divergent regulation of terpenoid metabolism in the trichomes of wild and cultivated tomato species. Plant Physiol..

[63] Mukrimin M., Kovalchuk A., Ghimire R.P., Kivimäenpää M., Sun H., Holopainen J.K., Asiegbu F.O. (2019). Evaluation of potential genetic and chemical markers for Scots pine tolerance against *Heterobasidion annosum* infection. Planta.

[64] Keesey I.W., Jiang N., Weißflog J., Winz R., Svatoš A., Wang C.-H., Hansson B.S., Knaden M. (2019). Plant-Based natural product chemistry for integrated pest management of *Drosophila suzukii*. J. Chem. Ecol..

[65] Zhang Z., Yang T., Zhang Y., Wang L., Xie Y. (2016). Fumigant toxicity of monoterpenes against fruit fly, *Drosophila melanogaster*. Ind. Crops Prod..

[66] Priestley C.M., Williamson E.M., Wafford K.A., Satelle D.B. (2003). Thymol, a constituent of thyme essential oils, is a positive modulator of human GABA and a homo-oligosteric GABA receptor from *Drosophila melanogaster*. Br. J. Pharmacol..

[67] Houghton P.J., Ren Y., Howes M.J. (2006). Acetylcholinesterase inhibitors from plants and fungi. Nat. Prod. Rep..

[68] Finetti L., Roeder T., Calò G., Bernacchia G. (2021). The insect type 1 tyramine receptors: From structure to behaviour. Insects.

[69] Terhzaz S., Cabrero P., Brinzer R.A., Halberg K.A., Dow J.A., Davies S.A. (2015). A novel role of *Drosophila* cytochrome P450-4e3 in permethrin insecticide tolerance. Insect Biochem. Mol. Biol..

[70] Scanlan J.L., Gledhill-Smith R.S., Battlay P., Robin C. (2020). Genomic and transcriptomic analyses in *Drosophila* suggest that the ecdysteroid kinase-like (EcKL) gene family encodes the ‘detoxification-by-phosphorylation’ enzymes of insects. Insect Biochem. Mol. Biol..

[71] Baldwin S.R., Mohapatra P., Nagalla M., Sindvani R., Amaya D., Dickson H.A., Menuz K. (2021). Identification and characterization of CYPs induced in the *Drosophila* antenna by exposure to a plant odorant. Sci. Rep..

[72] Wang Q., Xu P., Sanchez S., Duran P., Andreazza F., Isaacs R., Dong K. (2021). Behavioral and physiological responses of *Drosophila melanogaster* and *D. suzukii* to volatiles from Plant essential oils. Pest Manag. Sci..

[73] Meng L.-W., Yuan G.-R., Lu X.-P., Jing T.-X., Zheng L.-S., Yong H.-X., Wang J.-J. (2019). Two delta class glutathione *S*-transferases involved in the detoxification of malathion in *Bactrocera dorsalis* (Hendel). Pest Manag. Sci..

[74] Tian F., Li C., Wang Z., Liu J., Zeng X. (2019). Identification of detoxification genes in imidacloprid-resistant Asian citrus psyllid (Hemiptera: Lividae) and their expression patterns under stress of eight insecticides. Pest Manag. Sci..

[75] De Souza M.T., de Souza M.T., Bernardi D., de Melo D.J., Gorgatti Zarbin P.H., Cassilha Zawadneak M.A. (2021). Insecticidal and oviposition deterrent effects of essential oils of *Baccharis* spp. and histological assessment against *Drosophila suzukii* (Diptera: Drosophilidae). Sci. Rep..

[76] D’Addabbo T., Avato P. (2021). Chemical composition and nematicidal properties of sixteen essential oils—A review. Plants.

[77] Renkema J.M., Wright D., Buitenhuis R., Hallett R.H. (2017). Plant essential oils and potassium metabisulfite as repellent for *D. suzukii* (Diptera: Drosophilidae). Sci. Rep..

[78] Ajayi O.E., Appel A.G., Fadamiro H.Y. (2014). Fumigation toxicity of essential oil monoterpenes to *Callobruchus maculatus* (Coleoptera: Chrysomelidae: Bruchinae). J. Insects.

[79] Allsopp E., Prinsloo G., Smart L.E., Dewhirst S.Y. (2014). Methyl salicylate, thymol and carvacrol as oviposition deterrents for *Frankliniella occidentalis* (Pergande) on plum blossoms. Arthropod-Plant Interact..

[80] Damtie D., Mekonnen Y. (2021). Toxicity and oviposition deterrent activities of thyme essential oils against *Anopheles arabiensis*. Psyche A J. Entomol..

[81] Marchetti E., Alberghini S., Battisti A., Squartini A., Baronio P., Dindo M.L. (2009). Effects of conventional and transgenic *Bacillus thuringiensis* galleriae toxin on *Exorista larvarum* (Diptera: Tachinidae), a parasitoid of forest defoliating Lepidoptera. Biocontrol Sci. Technol..

[82] Tan K.H., Nishida R. (2012). Methyl eugenol: Its occurrence, distribution, and role in nature, especially in relation to insect behavior and pollination. J. Insect Sci..

[83] Jankowska M., Łozowicka B., Kaczyński P. (2019). Comprehensive toxicological study over 160 processing factors of pesticides in selected fruit and vegetables after water, mechanical and thermal processing treatments and their application to human health risk assessment. Sci. Total Environ..

[84] Gaire S., Zheng W., Scharf M.E., Gondhalekar A.D. (2021). Plant essential oil constituents enhance deltamethrin toxicity in a resistant population of bed bugs (*Cimex lectularius* L.) by inhibiting cytochrome P450 enzymes. Pestic. Biochem. Physiol..

