# Supplementary material for: Monarda didyma Hydrolate Affects the Survival and the Behaviour of Drosophila suzukii
Source: Insects. 2022 Mar 11;13(3):280. doi: 10.3390/insects13030280 (PMC8955400; doi:10.3390/insects13030280)
Supplement: Supplementary file 1 [file insects-13-00280-s001.zip › insects-1624679-supplementary.pdf]

Table S1. Primer used in this study.

Gene name	Primer sequence (5'-3')	Melting temperature (°C)	Amplicon dimension (bp)
Cyp4e3	F: CAAAGCATCTGGAGCACATTT<br>R: GCCATTTGCTGCCATAACTC	62	117
Cyp4g15	F: CATCGAGCTCTTCAACGAGAA<br>R: CCATCGCAGTCTCCAATAGAAT	62	128
Cyp6a17	F: AAGACCAACGAGAAGCGAAAC<br>R: CTCGAAACCAGCCACAAAGA	60	220
Cyp6d5-2	F: GCTGACCTACGATGCCATATC<br>R: AGGTGTGCCCTTCTTGATTAC	60	154
GstZ2	F: GCGATGAACCTGAAGGAGATAC<br>R: AGGGTGTGTCCATCGATCT	62	140
GstD10	F: GCGTGCTATCATGGTGTATCT<br>R: TCTTGTAAAGGGTGCCCATATC	62	143
Est1	F: TTGGGCGCCAAGGTTATT<br>R: GATTTCGCTGTCGTAGATGGT	62	129
Actin	F: CTACCACAACGATGCCAAGA<br>R: AAGGTCAGGAAGCCGAGA	61	180
TBP	F: CCACGGTGAATCTGTGCT<br>R: GGAGTCGTCCTCGCTCTT	61	182

Table S2. Comparison of probing, non-probing and dabbing phases (mean ± SEM) measured by EPG on *D. suzukii* females left for 24 h on both untreated and HY treated diets. n.s. = not significant at $p < .05$. * $p < 0.05$ according to non-parametric ANOVA Mann–Whitney U test.

**PROBING**	Mean probing duration per insect (mins.)	Mean number of probing events	Mean duration of probing event (mins.)
100% *M. didyma* HY	13.06 ± 1.88	156.70 ± 21.54	0.12 ± 0.04
Control	12.48 ± 2.22	157.91 ± 25.02	0.10 ± 0.02
Statistical analysis	n.s.	n.s.	n.s.

**NON-PROBING**	Mean non probing duration per insect (mins.)	Mean number of non-probing events	Mean duration of non-probing event (mins.)
100% *M. didyma* HY	106.73 ± 1.89	212.30 ± 26.32	0.69 ± 0.08
Control	107.29 ± 2.21	197.39 ± 30.37	0.90 ± 0.17
Statistical analysis	n.s.	n.s.	n.s.

**DABBING**	Mean dabbing duration per insect (sec.)	Mean number of dabbing events	Mean duration of dabbing event (sec.)
100% *M. didyma* HY	11.47 ± 2.46 sec	54.39 ± 10.22 sec	0.20 ± 0.01 sec
Control	10.17 ± 2.24 sec	37.83 ± 8.42 sec	0.27 ± 0.02 sec
Statistical analysis	n.s.	n.s.	*

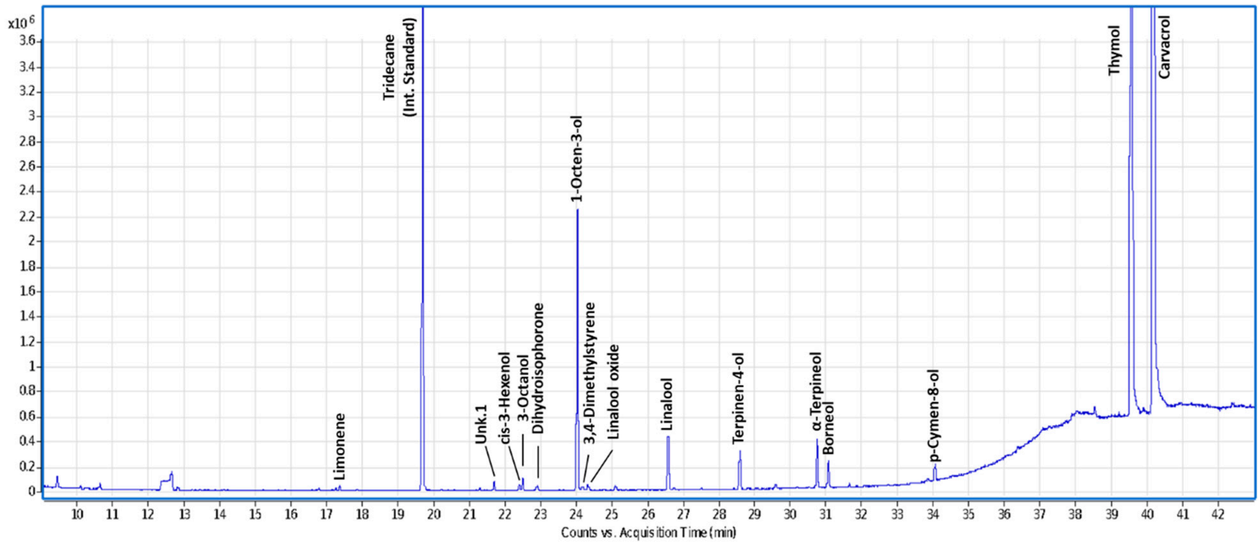

Figure S1. Magnification of Figure 2 of the total ion chromatogram (TIC) of main monoterpenes and other volatile organic compounds present in *M. didyma* HY.
